# Vestibular function in children with cochlear implant: Impact and evaluation

**DOI:** 10.3389/fneur.2022.938751

**Published:** 2022-08-23

**Authors:** Jianhang Deng, Qianchen Zhu, Kangjia Zhang, Dinghua Xie, Weijing Wu

**Affiliations:** Second Xiangya Hospital, Central South University, Changsha, China

**Keywords:** cochlear implant, vestibular function, children, malformation, recommended strategies

## Abstract

Over the last 30 years, cochlear implant (CI) has been dedicated to improving the rehabilitation of hearing impairments. However, CI has shown potential detrimental effects on vestibular function. For children, due to atypical symptoms and difficulty in cooperating with vestibular function tests, systematic and objective assessments of vestibular function with CI have been conducted sparsely. This review focuses on the impact of vestibular function in children with CI and summarized the evaluation of vestibular function in children. In addition, some recommended strategies are summarized and proposed.

## Introduction

In the last decades, children with bilateral severe to profound sensorineural hearing loss (SNHL) have benefited greatly from cochlear implants (CI). While the effects of CI surgery on residual cochlear function have been studied more frequently, its effects on vestibular function have received less attention.

Clinical studies have shown a 2–35% incidence of vertigo and a 20–80% incidence of vestibular abnormalities in postoperative CI patients (1). Some scholars believe that patients with CI will develop symptomatic or asymptomatic vestibular impairment sooner or later (2).

CI can cause vestibular impairment by direct damage to vestibular sensory structures, disruption of fluid balance in the inner ear, inflammatory response, or direct electrical stimulation, according to Handzel et al. (3). The normal motor development of newborns and children, as well as optimal motor skill in preschool and school-age children, is dependent on the activity of the inner ear balance organ (vestibular organ). When compared to children without vestibular dysfunction, children with vestibular dysfunction have delayed development of gross motor milestones such as standing and walking (4). We firmly believe that vestibular examination is critical for patients because of its impact on their early development. Vestibular function testing in children can be challenging for a variety of reasons. Children have limited communication skills, atypical symptoms, difficulty recognizing their symptoms are abnormal, short attention span, and sometimes nausea and vomiting reactions (5). Various clinical strategies for adapting vestibular testing to children have been proposed since the 1980s. The most commonly reported approaches to date are minimized caloric irrigations, adapted rotational tests, video-assisted head impulse test techniques (vHIT), and vestibular evoked myogenic potentials (VEMP) (6).

In this paper, we analyze the factors that cause changes in vestibular function after CI surgery and summarize the assessment methods of vestibular function and vestibular rehabilitation patterns in children with CI. Meanwhile, we explore the outcomes of CI on vestibular function and the selection of corresponding diagnosis and treatment strategies.

## Impact of vestibular function in children with cochlear implant

### Mechanisms of vestibular function changes due to cochlear implant surgery

Several mechanisms that cause vestibular impairment have been reported, damage to the vestibular end organs may occur as a result of direct trauma caused by possible misalignment of the electrode insertion into the vestibular steps, electrical stimulation of the vestibular organs by the implant, intraoperative perilymphatic deficit, endolymphatic flow disturbance generating endolymphatic edema, and foreign body reaction with labyrinthitis or vestibular fibrosis (7–10). Histopathological studies of the temporal bone after cochlear implantation show cochlear effusion with collapsed vesicles in more than half of the cases (3). Another common finding is the incorrect insertion of electrode arrays in the vestibule (11).

Because of its proximity to the electrode insertion path, the saccule is thought to be more vulnerable to damage than the utricule or semicircular canal, and this proximity may make the saccule more susceptible to surgical injury in the presence of electrode insertion, drilling, or changes in the fluid environment of the inner ear (12, 13).

Different surgical accesses have also been reported to have different effects on vestibular function. Yoon et al. (14) divided the patients into two groups according to surgical access: the cochleostomy group and the round window implant group. Patients in the round window implant group had a lower risk of postoperative vertigo (5.5%) than those in the cochleostomy group (8.8%). Several other studies have shown the round window approach was more beneficial to the protection of vestibular function (15). However, the results of different studies on the surgical approach are currently mixed, and more relevant studies are still needed.

### Symptoms of vestibular hypofunction in children

Visual abnormalities, torticollis, clumsiness, recurrent vomiting, episodic spontaneous bouts of dizziness, vegetative lethargy, otalgia, headache, ataxia, lack of postural control, delay in gross motor skills, or learning or reading impairments all common symptoms of vertigo in children (16). In the pediatric population, vertigo and imbalance are frequently underestimated or ignored. Firstly, Children have difficulty expressing abnormal sensations of vertigo, dizziness, or imbalance, adding a layer of complexity to the diagnosis. Secondly, the differential is complicated and distinct from adult vertigo, making it difficult to diagnose. Thirdly, children can usually adapt very quickly and compensate for the lack of vestibular loss, probably as a result of their neuroplasticity and the stability of other sensory input systems, resulting in a short and insignificant duration of symptoms (17).

Besides, in the majority of studies, self-perceived symptoms of vertigo often differ from objective test results. One reason is that most studies have used only one or a few methods of vestibular function detection, making it difficult to analyze the full complexity of the vestibular apparatus as no single vestibular function examination can provide a complete assessment of vestibular function. Another explanation is that while objective testing shows vestibular impairment, central compensation may reduce vertigo symptoms, or that vestibular organ damage is too mild to be detected. In addition, it has also been suggested that psychological factors such as anxiety and depression may be responsible for the disagreement (18).

Consequently, children's vertiginous complaints are often overlooked, erroneously diagnosed as clumsiness, or attributed to behavioral disorders (16). A complete history, standardized questionnaires, routine physical examination, imaging, and even more advanced tests are all required for the diagnosis of pediatric vestibular disorders (19).

### Factors influencing postoperative changes in vestibular function

The following factors may be associated with the development of postoperative vertigo: (1) gender; (2) age; (3) surgical side selection; (4) electrical stimulation; (5) inner ear malformation; (6) structural factors

#### Gender

Data on the relationship between gender and auditory vestibular disorders remain inconclusive. Clinical data suggest that gender may be a potential causative factor in auditory vestibular disorders. Anatomical differences in the inner ear exist between males and females, and physiological and/or hormonal influences between the sexes can produce different clinical findings on audiological and vestibular tests. Besides, Meniere's disease, BPPV, and other vestibular disorders are considered to be related to estrogen levels (20).

#### Age

Age diversity may cause differences in the results of various vestibular tests.

A meta-analysis showed that only 1.7% of children with CI had postoperative vertigo, compared with 31.3% of patients in the adult CI population (1). The reason for this may be that the child's vestibular function is more compensatory, or the child may not be able to represent their symptoms. The immaturity of central inhibitory vestibulo-ocular reflex regulation, cerebellar control, central vestibular adaptation, and visual-vestibular interactions may explain the much greater levels of rotational gain in children compared to adult normative data (21, 22).

For cVEMP, when pediatric data were compared to adult standards, it was discovered that children had significantly shorter P1 and N1 latencies and larger interpeak amplitudes (23) which could be explained by structural differences (24, 25). Besides, it was reported that N1 latency was significantly positively correlated with age, whereas the threshold parameter was significantly negatively correlated (5). While according to Picciotti et al. (26), no age trends were observed for latency parameters. Kelsch et al. (23) also covered a significant N1 latency extension. These differences may be partly related to differences in test protocols and the equipment used.

Toward the rotatory test, different studies have reached opposite conclusions. Charpiot et al. (27) reported decreasing gain values with increasing age. However, Maes et al. (5) showed no discernible age trends were remarkable for the rotatory test.

There are conflicting findings regarding whether the increase in vHIT in children varies with age. Lower gain values have been observed in children under the age of three, with a quick increase in vHIT gain up to the age of six, and then a slower increase up to the age of sixteen. In addition, with age, the variability of vHIT gain decreases (28). From the age of sixteen, vHIT increase appears to remain stable until the eighth or ninth decade, when it begins to fall (29–31).

The effect of age on vestibular function is inconclusive and still needs further study.

#### Bilateral and contralateral cochlear implant

As the use of bilateral implants grows, it will be critical to understanding the effects of bilateral CI surgery on the vestibular system, which will be beneficial to both the CI team and the patient. Guan et al. (32) believes no remarkable differences in the abnormality rate between children with first- and second-sided CI implantation 1 month after CI, demonstrating that the effects of unilateral and bilateral sequential CI on vestibular function are similar. The VEMP, on the other hand, revealed that children implanted with a second-side CI had a higher rate of anomalies than children implanted with a first-side CI. This might be explained by the ceiling effect, as these children's vestibular functions had already been harmed by the initial CI. In the case of vHIT, no statistically significant difference in abnormality rates existed before and after implantation, which was in line with earlier findings (33). According to Abouzayd et al. (34) no significant differences in aberrant rates after CI were found between first-side CI-implanted adults and children, or between first- and second-side CI-implanted children, implying that vestibular function abnormalities caused by CI surgery may be independent of age at CI and CI access (unilateral or sequential bilateral).

There were no significant variations in DHI and PVSQ ratings between adults and children pre-and post-implantation for unilateral CI, according to the vertigo questionnaire (32). PVSQ scores in children with bilateral CI were significantly higher on day 3 after implantation but significantly lower on day 30, indicating that these changes could be due to initial postoperative response to anesthesia or a middle/inner ear injury (32).

Das et al. (35) reported that bilateral cochlear implantation may offer extra benefits for vestibular function and is safe, with little risk when compared to unilateral implantation. Meanwhile, Dhondt et al. (36) suggested that CI had modest effects on vestibular function in children. As a result, the various benefits of bilateral implantation at the same time may outweigh the risk of postoperative vestibular impairment. In some situations, sequential bilateral implantation may be required since the impact on vestibular function can be influenced by a range of circumstances, including surgical manipulation, inner ear deformity, and so on. When deciding whether to conduct simultaneous or sequential bilateral CI, consider factors such as vestibular function.

To completely assess the hazards of bilateral cochlear implantation on the vestibular system, more research with long-term follow-up is needed.

#### Electrical stimulation

Parkes et al. (37) found that cochlear current stimulation can produce vestibular potentials in patients, suggesting that cochlear currents can spread from the cochlea to the vestibule (37). Gnanasegaram et al. (38) reported that about half of the children with CI who had vestibular deficits had spatial disorientation, but that this perceptual deficit was corrected by the current from the cochlear switch-on, possibly because (1) the current stimulation increased vestibular nerve activity and (2) the center could use the electrical stimulation from the cochlea as a supplement to vestibular stimulation. Electrical stimulation of the CI device has been demonstrated to affect VEMP responses in several studies (39).

Furthermore, Xu et al. (40) hypothesized that electrical stimulation of the CI could influence both ipsilateral and contralateral responses. When the device is turned on, electrode stimulation may alter the central vestibule to account for changes in contralateral cVEMP amplitude. Others speculated on the possibility of hyperexcitability as a result of electrical stimulation (41).

Research on the effects of electrical stimulation on vestibular function is insufficient and needs more attention.

#### Inner ear malformation

Congenital inner ear malformation (IEM) is a group of diseases that cause structural abnormalities in the inner ear due to developmental disorders at different stages of embryonic life. It is one of the common causes of congenital sensorineural deafness, with a group incidence rate of 1/2,000 to 1/6,000 (42). According to Jensen (43), 20% of children with congenital sensorineural hearing loss (SNHL) will have an inner ear defect. Among the malformation of bone labyrinth in the inner ear, the large vestibular aqueduct syndrome (LVAS), common cavity deformity (CCD), and Mondini malformation are more common (42). IEM was initially considered a contraindication to CI, but now CI has helped many IEM patients improve their hearing.

##### Large vestibular aqueduct syndrome

Sensorineural deafness is a symptom of large vestibular aqueduct syndrome, a congenital abnormality of the inner ear. Wang et al. (44) looked at vestibular function in kids with LVAS and kids with normal CT performance. They discovered that in children with normal CT, the overall VEMP abnormality rate increased significantly from pre to post ci, however in children with LVAS, there was no significant change in the overall VEMP abnormality rate. The findings imply that the effects of CI on otolith function differ between children with LVAS and those with normal CT. The pressure created during electrode insertion in children with LVAS can be discharged or released into the endolymph fluid *via* the larger vestibular aqueduct with less injury. Zhou et al. (45) evaluated vestibular function in LVAS patients, and the results of VEMPs suggested hyperactive vestibular function while the Caloric Irrigation suggested hypoactive vestibular function, the inconsistency needs to be evaluated comprehensively.

##### Common cavity deformity

Common cavity deformity (CCD) accounts for 25% of IEM cases (46). “A cystic cavity resembling the cochlea and vestibule, but without demonstrating any differentiation into cochlea and vestibule,” is how the CCD is defined (47). With this pathology, the patient may have normal, narrow, or wide internal auditory canals (IAC). According to McElveen et al. (48), patients with CCD rely on their visual and somatosensory systems rather than much vestibular input. Therefore, despite the fact that the entrance site corresponds to the lateral semicircular canal, it is unlikely that patients will have vertigo or dizziness after CI. Due to deformed inner ear architecture, the facial nerve typically follows an abnormal path in CCD, and the round window may not be apparent (49). The most likely cause of postoperative nystagmus is direct stimulation of the vestibular branch and after 3 months, this phenomenon exhibited adaptability. Three-dimensional TSE MRI is essential in the demonstration of cochlear and vestibular divisions of cochleovestibular nerve (49).

##### Mondini malformation

Mondini's dysplasis (MD), is brought on by a developmental stop in the seventh week of pregnancy. Currently, it is categorized as incomplete partition type 2 (IP-2) and is distinguished by 1.5 turns of cochlea with a normal basal turn, cystic apex caused by merged middle and apical turns, enlarged vestibular aqueduct, and dilated vestibule (50–52).

Patients with MD have been found to experience degenerative alterations in their vestibular system and have underdeveloped vestibular sensory organs and an enlarged vestibular aqueduct is one of the inner ear anomalies connected to Mondini dysplasia that is most typical (53). In Kaya's study (54), 44% of patients with MD had aplastic semicircular canals and the loss of spiral ganglion cells was either severe or mild in the temporal bone samples with semicircular canal anomalies. The loss of vestibular type I and type II hair cells they observed in the MD group was statistically significant in all semicircular canals. This fact should be taken into account when evaluating cochlear implants.

##### Structural factors

Vestibular dysfunction is often present in children with deafness, and the literature reports a prevalence of 20–70% (55, 56). Alexandra (57) believes that the degree of vestibular function impairment is related to the degree of hearing loss. Wolter et al. (58) found that children with unilateral deafness also develop balance deficits, hypothesizing that the “auditory preference syndrome” caused by unilateral deafness and the lack of symmetrical auditory stimulation in the brain may be responsible for the balance deficits.

## Evaluation of vestibular function in children

There are fewer studies on the assessment of vestibular function in children, mainly because children have difficulty describing vertigo symptoms and cooperating with vestibular function tests. It is necessary and important to have an appropriate assessment of vestibular function in children.

### Pediatric vestibular function questionnaire

Vertigo and vestibular dysfunction in children can cause several symptoms. Furthermore, youngsters may not be able to fully express their symptoms. As a result, clinicians can use an appropriate questionnaire to measure the degree and impact of dizziness or vestibular loss (59). There are limited studies using questionnaires for pediatric populations, even though there are many questionnaires used to assess vestibular symptoms in adults.

Pavlou et al. (59) designed the PVSQ to accurately assess the severity of vestibular symptoms in children with vestibular symptoms. The test has “excellent accuracy” in distinguishing the presence or absence of abnormal levels of dizziness and/or instability. In 95% of children, the optimal cut-off score correctly identified abnormal levels of vestibular symptoms and accurately reported them in 85% of healthy children. The PVSQ provides the physician with a preliminary understanding of whether the child has symptoms related to vestibular hypofunction, and is also essential for screening children for vestibular function.

### Motor development and balance

Initial understanding of motor development can be obtained by knowing when the child lifts his head, sits crawls, and walks, as well as some motor development schedules. The balance test portion of the Bruininks-Oseretsky Test of Motor Proficiency Second Edition (BOT-2) is a generally used balance assessment method.

Adequate attention should be given to the child's motor development and balance.

### Vestibular function test

Assessment of vestibular function includes otolithic and semicircular canal function.

#### Otolithic function

Otolith organs include the saccule and utricle. VEMPs can reflect the functional status of the saccule and utricle. The evaluation of the results includes threshold, wave amplitude, latency, etc. VEMPs are muscle responses elicited by sound, electric current, or bone-conducted vibration stimulation of the vestibular end organs.

The cVEMP recorded from the sternocleidomastoid muscles and the oVEMP recorded from extraocular muscles have both been characterized. To put it another way, cVEMP is a test for saccular (inferior vestibular nerve) otolith functions, while oVEMP is for utricular (superior vestibular nerve) otolith functions (60). The vestibular nerve assessment approach cVEMP is now the most widely utilized to examine the effects of CI on vestibular function in children. This could be related to cVEMP's simpler fit and the fact that, because of its proximity to the CI insertion site, the balloon is thought to be the most vulnerable to surgical effects. Furthermore, VEMPs are straightforward to assess and work with for younger children.

When assessing extremely young children, particularly newborns under the age of 6 months, cVEMP is especially significant because the VOR is naturally faulty in these youngsters. The cVEMP can be examined in a child's supine posture (<15 months) or in a child's sitting upright position to generate cervical muscle activation through head suspension or continuous rotation. Owing to the many procedural biases that can arise in VEMP recordings, there is a danger of erroneous pathological findings (not due to a lack of response due to vestibular insufficiency). Therefore, Verrecchia et al. (6) consider the absence of VEMP responses as true vestibular insufficiency in at least three consecutive trials.

When peripheral vestibular nerve involvement is suspected, VEMP testing of the vestibule in children is advised. In newborns, cVEMP testing is possible. However, oVEMP pathway does not mature until 3 years of age and reaches amplitudes and latencies similar to those of adults. Janky et al. (61) recommend that all children at age 3 should complete oVEMP testing.

#### Semicircular canal function

##### Video head impulse test

The vestibulo-ocular reflex (VOR) arising from the three semicircular canals is measured using the video Head Impulse Test (vHIT). As a result, vHIT also evaluates both branches of the vestibular nerve; posterior canal vHIT is an assessment of the inferior portion of the vestibular nerve and anterior and horizontal canal vHITs are assessments of the superior portion of the vestibular nerve (61). The vHIT (together with VEMPs) is a relatively recent assessment approach that provides a quick, objective vestibular exam ideal for youngsters. As a result, it's ideal for a follow-up evaluation in the pediatric population (6). The vHIT is the optimum approach for testing canal function, according to Verrecchia et al. (6).

Lower gain values have been observed in children under the age of three, with a quick increase in vHIT gain up to the age of six, and then a slower increase up to the age of sixteen. When defining standard values for children, age-related changes in vHIT gain should be taken into account, as should the possibility of corrective sweep when vHIT is regarded abnormal.

VHIT has several advantages. Firstly, it can independently assess the functional status of the six semicircular canals. Secondly, it is the only test that can assess the function of the anterior and posterior semicircular canals. Thirdly, during high-frequency stimulation, it can respond to the function of the semicircular canals. Furthermore, regardless of the condition of the middle ear, including the existence of pressure equalization tubes, perforations, or a mastoid cavity, vHIT can be performed (61).

However, due to visual fixation and VOR development in children under 6 months of age, false-positive results may be obtained during evaluation (6). This factor should be taken into account when performing vHIT on children.

##### Caloric irrigation

Caloric irrigation uses a temperature gradient to test the horizontal semicircular canal and the inferior branch of the vestibular nerve. The mini ice-water caloric test (mIWC) is an altered version of an ice-water caloric method that was previously proposed.

The time spent in cold water was reduced by 10 s, yet there was enough of a temperature gradient (water temperature ≤ 10°C) to guarantee optimal vestibular activation. In comparison to other ways previously tested, this method significantly increased the child's cooperation (62, 63). Only when caloric nystagmus was absent on two test repetitions was it thought to be indicative of vestibular insufficiency.

Caloric irrigation offers low-frequency information about the superior branch of the vestibular nerve and the horizontal semicircular canals that are particular to the ear.

By 6 to 12 months, the caloric response in newborns is assumed to be mature, and the likelihood of having a normal response improves as children gain weight. The magnitude of slow-phase velocities in response to caloric stimulation declines with age in children aged 2–10 (64).

However, this test can cause dizziness and nausea, so it is often difficult for children to tolerate. Due to a certain fear of the child during the examination and discomfort, the degree of cooperation is poor.

##### Rotary chair

Rotary chair is a midfrequency (0.01–0.64 Hz) assessment of the horizontal canal and superior branch of the vestibular nerve. Cushing et al. (56) concluded that the swivel chair test is the test of choice for suspecting vestibular damage in children because that can be used at any age and is easy to match. Reduction in VOR gain is the best predictor of kinesthetic imbalance. Rotary chair testing is commonly used to assess overall vestibular reactivity and is particularly useful in detecting bilateral vestibular loss and determining the severity of bilateral vestibular loss. It should be noted that middle ear effusion can affect the swivel chair reaction, therefore, it is recommended to use the swivel chair test for tympanometry (65).

Noteworthy, because these tests examine diverse structures of the vestibular system, there may be some disagreement between otolaryngologic and otolithic testing. This divergence may be because the test partially damaged inner ear organs with different levels of function at different test sites. Therefore, the otolith/ear canal inconsistency is interpreted as a reduced vestibular function rather than full vestibular dysfunction (6). Besides, the divergence between vHIT and mIWC also occurs when subjects have inner ear malformations.

In conclusion, caloric testing is frequently not an option for examining vestibular function in youngsters due to tolerance or time constraints. The swivel chair test is considered the gold standard for diagnosis in patients with bilateral vestibular injuries (66). The chair test and vHIT results, on the other hand, may not be consistent.

In patients with severe bilateral vestibular loss, Judge et al. (66) discovered higher agreement between rotary chair and vHIT. While vHIT showed a pattern consistent with unilateral vestibular loss in 25% of individuals with bilateral vestibular loss, rotary chair showed a pattern consistent with bilateral vestibular loss. The degree of vestibular loss might vary in youngsters, where vHIT and rotary chair are the key measures. It is suggested that vHIT is a sufficient first-level evaluation. Rotary chair test is not required if the vHIT findings are abnormal. If rotary chair test is normal, it can aid in the detection of additional signs of vestibular loss.

Mild vestibular loss has no effect on vHIT or rotary chair. vHIT and rotary chair abnormalities are often not present until caloric weakness surpasses 40–45% in the case of unilateral weakness. Caloric testing can be used to rule out moderate, unilateral vestibular loss when vestibular involvement is indicated and the swivel chair and/or vHIT are normal (67).

## Recommended strategies

### Pre-operative

Surgeons need to take a detailed history and complete imaging studies. The feasibility of performing bilateral and contralateral CI is fully evaluated preoperatively, and vestibular function must be taken into account as an important consideration.

#### Vestibular screen

A large-scale, safe and affordable vestibular test for newborns and infants is worth consideration as well as an early vestibular assessment in terms of cochlear implantation (CI). Documented vestibular failure may lead to the diagnosis of SNHL with vestibular failure in the clinical setting, where up to 35% of patients with congenital SNHL (sensorineural hearing loss) do not have a precise diagnosis. This is especially common in disorders such as inner ear abnormalities (61).

For all children experiencing dizziness, a vestibular evaluation is suggested. Furthermore, because of the high prevalence of vestibular loss in children with SNHL, the vestibular loss should be considered when hearing loss is suspected. Clinicians can utilize a vestibular screen to see if a kid has vestibular impairment and if more testing is needed.

The modified clinical test of sensory integration on balance, the bedside head thrust test, the Emory clinical vestibular chair test, the dynamic visual acuity test, single-leg stance, tandem standing, age of gross motor attainment, and severity of hearing loss have all been recommended as screening measures for children with hearing loss (61). Besides, the excellent feasibility of VEMP coupled with the newborn hearing screen program was proven by Verrecchia et al. (68). VEMP measurements could be completed in the majority of examined ears (86%) and in up to 97% of recordings with pre-stimulus EMG within the reference in the second step of hearing screening or after the clinical ABR. Furthermore, more than three-quarters of the trials yielded a clearly visible VEMP response, with the percentage rising to 91.5 percent when the test was performed under optimum clinical conditions.

Screen tests should be economical, simple to administer, benign, and cover populations with a high prevalence of the target condition, in addition to being diagnostic in nature (69). VEMP was recently included as a secondary vestibular nerve examination for all neonates with SNHL found in a hearing screen program in a large multicenter nationwide study (70). For all children who are evaluated following the first phase of the hearing test, it is advised that VEMP be incorporated into the hearing screen program (68).

In addition, parental worries about the degree of gross motor delay, sitting and walking delays, and hearing loss in children with hearing loss are markers of vestibular loss. These characteristics can be very useful in determining whether a child has bilateral vestibular loss. As a result, these indicators should be incorporated into screening tools for children with hearing loss (71).

To establish the diagnostic accuracy of VEMP as a sort of vestibular screen in children, more research is needed.

#### Vestibular prediction

The high prevalence of vestibular damage is linked to specific etiologies of hearing loss; for example, in some etiologies, such as meningitis, vestibular damage occurs in practically all patients, whereas other etiologies have varied effects on vestibular function (56).

Because of the enormous impact of vestibular loss on big muscle motor development and other outcomes, as well as the benefits of early management, it is critical to detect children with vestibular loss as soon as possible.

In early childhood, vestibular nerve injury is linked to the severity of hearing loss and motor impairments. Vestibular loss is thought to affect 30–74% of infants with severe hearing loss (72). According to reports, 50% of youngsters who are candidates for cochlear implants (CI) suffer from vestibular loss (56, 73).

In children with CI, vestibular damage is associated with an increased risk of falls and CI device failure (74). Hearing loss is more severe in children with bilateral vestibular impairment compared to children with normal or unilateral vestibular impairment (75).

Janky et al. (71) show that in a younger group, particular characteristics can predict vestibular loss. Vestibular loss was predicted by age to sit, age to walk, bilateral PTA, and parental worries about gross motor developmental delay. These four indicators may assist evaluate whether or not a kid with hearing loss need vestibular testing. According to ROC analysis, employing a threshold of 7.25 months for age-to-sit and 14.5 months for age-to-walk for detecting vestibular loss provides reasonable sensitivity and specificity (71). According to ROC analysis, employing a bilateral PTA cutoff of 40 dB has excellent sensitivity (80%) and a bilateral PTA cutoff of 66 dB has excellent specificity (91%). These indicators are more sensitive in detecting children who have suffered bilateral vestibular damage.

A vestibular test should be undertaken for children who have a hearing loss of more than 66 dB, particularly those who sit later than 7.25 months or walk later than 14.5 months, or whose parents have concerns about gross motor development (71).

#### Vestibular function test

Patients with severe dizziness disorder are at risk for social isolation, anxiety, depression, falls, and injury, hence identifying these patients with vestibular hypofunction before surgery is crucial (18). By knowing the side of vestibular insufficiency, preoperative vestibular assessment can play a role in minimizing the risk indications for CI, considering the potential vestibular impairment effect of CI (76), CI can be reconsidered when only the functional vestibular side is suggested. If unavoidable, parents can be informed about the possible risk of motor proficiency sequelae and reminded them the possibility of early adaptive intervention.

### Intra-operative

Appropriate surgical manipulation can reduce vestibular damage. The optimal surgical route for cochleostomy has been explored to minimize damage to the inner ear structures and the round window approach is recommended. Studies have shown that the anterior inferior/inferior way to cochleostomy, where the hole is drilled from below toward the round window annulus and gradually advanced toward the lower surface of the cavity, causes the least damage to the cochlea (77–79).

Inner ear damage induced by electrode insertion is linked to the size and shape of the CI electrodes utilized, in addition to the surgeon's surgical approach. Inner ear injury is more common with large-diameter and straight electrodes than with thin and curved electrodes. The use of “soft surgery” techniques in cochlear implantation is also considered to be beneficial in preserving residual hearing and balance function. Coordes et al. (80) proposes that ensuring that the electrode is fixed in the tympanic step reduces the incidence of postoperative vertigo. Slow insertion of electrodes and intraoperative topical application of corticosteroids may have a protective effect on vestibular function (81). While the depth of electrode insertion does not affect the postoperative vestibular function test (82). Undeniably, the insertion of CI electrodes requires even more attention in the case of anomalous or abnormal anatomy (7).

Exolymphatic fluid leakage can cause vertigo symptoms, and conical electrodes may prevent fistula-related symptoms, while Dania (83) believes that restabilization of electrodes may alleviate postoperative vertigo.

### Post-operative

Many factors affect vestibular function after CI surgery. With increasing attention to changes in vestibular function after CI surgery, more and more treatments have been proposed.

#### Vestibular rehabilitation

Postoperative vestibular function rehabilitation is plastic and can be improved through training or corresponding treatment. Vestibular function can be improved through training or treatment, but it is difficult to fully recover. Saki et al. (84) studied 21 patients with vertigo and balance disorders after CI surgery, who underwent vestibular rehabilitation. The DHI and VAS were performed at weeks 1, 2, and 4, with the results that the DHI and VAS scores at weeks 2 and 4 were significantly better than those of the control group, indicating that vestibular rehabilitation had a positive effect on the vestibular symptoms of the patients receiving CI. Magdalena et al. (85) performed preoperative and postoperative vestibular function examinations in 55 CI patients with low-frequency residual hearing, and all of them underwent postoperative electroacoustic stimulation (EAS). The results showed an injury rate of 15.79% for the saccule, 19.04% for the utricle, and a decrease of 15.79% for the horizontal semicircular canals response, which is an average decrease of about 20% compared to the injury rate of the previous study. Compared to the previous study, concluded that EAS treatment was effective in improving vestibular function in patients with residual hearing.

#### Vestibular implant

The vestibular implant (VI) is comparable to a cochlear implant in that it captures motion rather than sound using a gyroscope (86). After that, the motion data is sent to a processor, which turns it into an electrical signal. Electrodes are then inserted near the ampullar branches of the vestibular nerve to transmit these electrical signals and stimulate the vestibular nerves (86). Motion information is transmitted to the brain in this way (87).

The Geneva-Maastricht group was the first to implant a completely working VI into a human, indicating that VI is feasible in humans (87). First, an electrically evoked vestibulo-ocular reflex could be elicited in the plane of the stimulated canal, and vestibular function could be partially restored in both low and high frequencies of movement (88, 89). Second, the brain can adjust to baseline inputs while continuing to respond to the implant's motor-induced conditioning (87). Third, the electrically evoked VOR had properties that were similar to those of natural VOR (90). Fourth, vestibulo-ocular and vestibulospinal reflexes can be elicited and recorded using vestibular evoked myogenic potentials and postural alterations, respectively (91).

Fifth, the VI's input perception varies: it's not always the sense of vertigo or spinning, but it can also be other sensations like sound or pressure (87). Sixth, residual natural vestibular information can be overcome by VI information when the brain performs non-linearly. In the case of fluctuating vestibular function, this could open the way for the VI to be used as a “vestibular pacemaker” (92, 93).

VI is a new treatment that uses direct cerebral nerve stimulation to treat bilateral vestibular lesions and other underlying vestibular illnesses.

Even though numerous hurdles remain in the development of the device and its implantation technology, the study reveals the feasibility and value of VI in improving clinical outcomes for individuals with certain vestibular illnesses who have failed to respond to standard treatments (86).

Future trials should validate this approach in a larger patient population.

#### Testing time

Different research on the impact of cochlear implant surgery on vestibular function varies widely from study to study and may depend on the lack of a standardized postoperative testing time.

Currently, there is no definite conclusion on the time of vestibular function evaluation after cochlear implantation. In some studies on vestibular function changes after cochlear implantation, the time of vestibular function evaluation varies from 1 month to 12 months after surgery (18, 94–96). Long-term follow-up is available to observe the long-term effects of cochlear implantation on vestibular function in children. It is generally believed that the results of vestibular function assessment can be attributed to the surgical procedure at 6–8 weeks after surgery, before the initial CI device activation, and therefore before the child has experienced electrical stimulation. Other studies have shown that vestibular function of patients deteriorates 3–6 months after cochlear implantation and tends to be stable about 14 months after implantation (18). Therefore, vestibular function evaluation can be conducted at different postoperative time points. At our center, vestibular function was studied at 1 day preoperatively and 1,6,12 months postoperatively.

## Conclusion

Although clinical researchers are now becoming aware of the importance of preserving vestibular function, vestibular function testing on children is still a relatively new area. Even though most children exhibit large and rapidly compensating sensory deficits, vestibular dysfunction cannot be ignored. When possible, screening of all patients requiring vestibular neurological examination is necessary, as is postoperative evaluation. Intraoperative thin and curved electrodes, “soft surgery” technique and round window placement may reduce vestibular dysfunction, but this remains to be proven.

In addition, vestibulo-cochlear implantation with artificial electrical stimulation of the vestibule through external electrodes similar to CI is a new technique to be explored for the treatment of patients with severe and very severe sensorineural deafness with persistent vestibular function. More research is needed to better guide the clinical application of CI and to provide optimal outcomes for CI implantation patients with optimal implantation and rehabilitation outcomes.

## Author contributions

JD and WW contributed to conception of the study and wrote the first draft of the manuscript. QZ and KZ wrote sections of the manuscript. DX supervised the manuscript. All authors contributed to manuscript revision, read, and approved the submitted version.

## Conflict of interest

The authors declare that the research was conducted in the absence of any commercial or financial relationships that could be construed as a potential conflict of interest.

## Publisher's note

All claims expressed in this article are solely those of the authors and do not necessarily represent those of their affiliated organizations, or those of the publisher, the editors and the reviewers. Any product that may be evaluated in this article, or claim that may be made by its manufacturer, is not guaranteed or endorsed by the publisher.
